# What’s in a Dream

**Published:** 1890-04

**Authors:** 


### WHAT'S IN A DREAM.

A case of telepathy was that of the son of Bishop Lee, of Canada. The bishop fell down a flight of stairs in his residence, receiving severe injuries, for which he was afterward treated at Hyde Park, near Chicago. At the instant of the accident his son was asleep in Denver. He sprang out of bed crying, "Father is falling." His wife told him he was dreaming, but he was so impressed that he telegraphed home and learned that his dream, or whatever it was, was a reality.

A story with a little romance in it, is that of S. R. W., of Bridgeport, Conn., who was returning from England on an ocean steamer. One night he dreamed that his wife, who was then in Bridgeport, opened the door of his state-room, looked hesitatingly in and then came forward and kissed him. When he awoke in the morning the man who occupied the upper berth in his state-room looked down and said: "You're a pretty fellow to let a woman come in here in the night and kiss you." Pressed for an explanation, he described the scene which he had experienced. Arrived at home, he was asked by his wife: "Did you receive a visit from me on such a night? I made you one. I was worried because of the reported storms that night. I dreamed I went out on the ocean and came upon a great black steamship. I went up the side and along the corridor and opened your door. I saw a strange man looking at me from an upper berth. I was afraid at first, but finally I stepped in and kissed you."—*St. Louis Globe Democrat.*

